# A case report of multiple system atrophy-mimics: Importance of comprehensive evaluation in suspected familial cases

**DOI:** 10.1097/MD.0000000000047266

**Published:** 2026-01-23

**Authors:** Chanhee Jeong, Matthew Farrer, Vikram Khurana, Han-Joon Kim

**Affiliations:** a Department of Neurology, Seoul National University Hospital and Seoul National University College of Medicine, Seoul, Republic of Korea; b Department of Neurology, University of Florida, Gainesville, FL; c Department of Neurology, Brigham and Women’s Hospital and Harvard Medical School, Boston, MA.

**Keywords:** case report, metachromatic leukodystrophy (MLD), multiple system atrophy (MSA), whole genome sequencing (WGS)

## Abstract

**Rationale:**

Multiple system atrophy (MSA) is primarily a sporadic neurodegenerative disorder, and a positive family history is considered against the diagnosis. While rare familial clusters are reported, they pose a significant diagnostic challenge. This report describes 2 cousins with phenotypically classic MSA who underwent genetic testing to investigate a potential shared etiology, leading to a diagnosis of a rare MSA mimic.

**Patient concerns:**

Case 1: a 77-year-old male presented with Parkinsonism (bradykinesia, resting tremor) and neurogenic orthostatic hypotension. Case 2: a 55-year-old female, case 1’s 1st cousin, presented with progressive limb ataxia, dysarthria, and neurogenic orthostatic hypotension. Both patients reported a poor therapeutic response to levodopa and a progressive decline in functional mobility.

**Diagnoses:**

Initially, case 1 was diagnosed with clinically probable MSA with predominant Parkinsonism, and case 2 was diagnosed with clinically probable MSA with predominant cerebellar ataxia (MSA-C). However, post-genetic analysis, case 1 was definitively diagnosed with late-onset metachromatic leukodystrophy (MLD). Case 2 remained clinically classified as MSA-C as no significant genetic variants were identified.

**Interventions:**

Clinical evaluation and levodopa trials were conducted for both patients. To investigate the suspected familial MSA cluster, whole-genome sequencing was performed for both individuals to identify shared pathogenic variants.

**Outcomes:**

Whole-genome sequencing identified biallelic pathogenic mutations in the arylsulfatase A gene in case 1, confirming MLD. No shared genetic etiology was found in case 2. The discovery of MLD in case 1 provided an alternative metabolic explanation for his symptoms, thereby refuting the initial hypothesis of a shared familial MSA link between the 2 cousins.

**Lessons:**

This case highlights that late-onset MLD can closely mimic the clinical phenotype of MSA. Clinicians should maintain a high index of suspicion when encountering familial MSA. A comprehensive genetic evaluation is essential in such cases to exclude metabolic or hereditary mimics before concluding a rare familial presentation of a typically sporadic synucleinopathy.

## 1. Introduction

Multiple system atrophy (MSA) is generally considered a sporadic disease while a positive family history is considered a red flag.^[1]^ Nonetheless, there have been reports of familial MSA cases with related genes, such as COQ2 gene, and some were neuropathologically-proven.^[2-4]^ Here, we present 2 first cousins who were both clinically diagnosed with MSA, but in whom subsequent genetic evaluation revealed an alternative diagnosis, refuting the initial suspicion of familial MSA.

## 2. Materials and methods

We conducted a retrospective review of 2 cases initially diagnosed with MSA based on clinical presentation. Both patients underwent thorough neurological examinations, brain imaging, and autonomic function tests. Given their familial relationship as first cousins, further evaluation was pursued to investigate the possibility of familial MSA. Whole-genome sequencing (WGS) was performed on both patients to identify potential genetic factors (Supplementary Digital Content 1, Supplemental Digital Content, https://links.lww.com/MD/R188; test methods, which demonstrates detailed WGS methods).

## 3. Case descriptions

**Figure 1.**
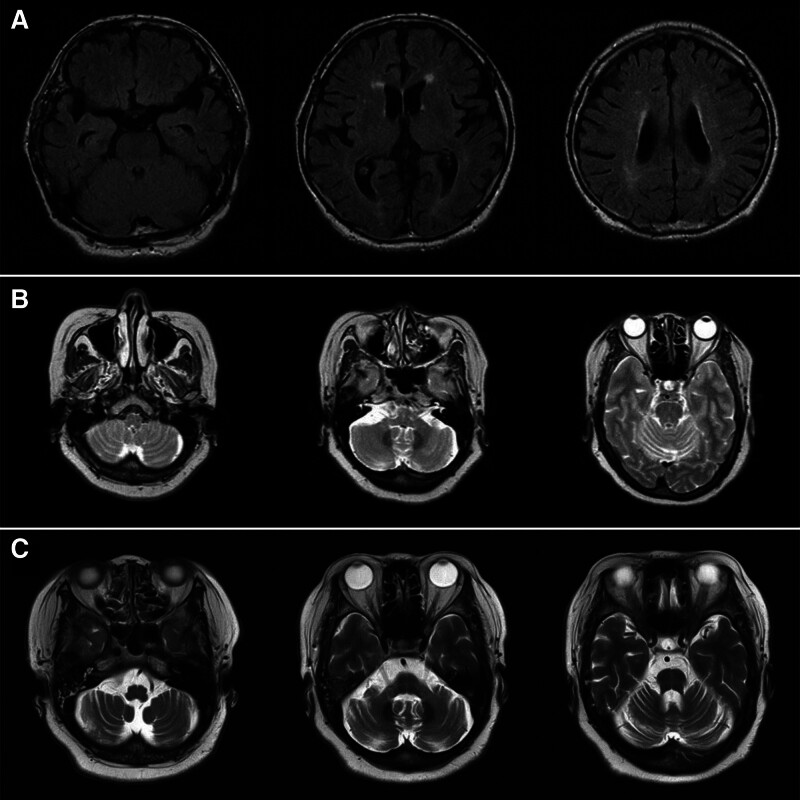
Brain MRI findings of the patients. (A) Case 1’s brain MRI T2 FLAIR axial images showing mild periventricular small vessel disease (age 77). (B) Case 2’s brain MRI T2 axial images showing mild diffuse cerebellar atrophy (age 52). (C) Case 2’s follow-up images showing progressed cerebellar atrophy and pontine atrophy (age 55). FLAIR = fluid-attenuated inversion recovery, MRI = magnetic resonance imaging.

A 77-year-old male (case 1) with underlying hypertension presented with progressive worsening of gait disturbance since age 76. His symptoms included resting tremor, urinary frequency and urgency, and frequent dizziness. Neurological examination revealed bradykinesia, mild postural instability and freezing of gait without evident ataxia and hypometric saccades. Autonomic function test revealed a blood pressure drop of 42/27 (mm Hg) within 3 minutes without heart rate compensation, indicative of neurogenic orthostatic hypotension. Ultrasonography showed 88 (cc) of residual urine after voiding, indicative of urinary retention. Brain MRI was unremarkable except for mild periventricular high signal intensity, which was attributable to underlying hypertension related small vessel disease (Fig. 1A). A levodopa trial of gradual dose increment up to 200 mg q.i.d. was unsuccessful and the patient’s gait disturbance and activities of daily living deteriorated on successive clinical visits.

A 55-year-old female (case 2) with underlying diabetes and dyslipidemia presented with progressive worsening of postural instability since age 52. Her symptoms included orthostatic dizziness, dysarthria, urinary frequency, incontinence and sleep-talking. Neurological examination revealed severe postural instability on pull test and shown definitive dysmetria on finger-to-nose and heel-to-shin tests. Autonomic function test revealed a blood pressure drop of 51/29 (mm Hg) within 3 minutes without heart rate compensation, indicative of neurogenic orthostatic hypotension. Brain MRI showed mild diffuse cerebellar and pontine atrophy at age 52 which had progressed at age 55 (Fig. 1B, C). A levodopa trial of 100 mg t.i.d. was unsuccessful and the patient clinically deteriorated on successive clinical visits with rapidly progressing dysarthria and dysphagia with aspiration tendency.

## 4. Diagnostic assessment

Diagnosis was based on the Movement Disorder Society MSA diagnostic criteria.^[1]^ Case 1, which presented with unexplained urinary incontinence, neurogenic OH, bradykinesia and resting tremor, fulfilled the core clinical features and rapid progression within 3 years was enough for a supportive clinical feature. Case 1 was diagnosed as clinically probable multiple system atrophy-parkinsonian type (MSA-P). In case 2, postural instability, neurogenic OH, gait and limb ataxia satisfy core clinical features and severe postural instability within 3 years was a supportive clinical feature. Case 2 was diagnosed as clinically probable multiple system atrophy-cerebellar type. There was no definite levodopa trial benefit, and no observations of downgaze palsy, dementia, hallucinations, fluctuating cognition, all of which could exclude the diagnosis.

However, the 2 patients were first cousins, with mother of case 1 being sibling of the father of case 2. Therefore, familial MSA was deemed possible and WGS was conducted in search for related genes (Supplementary Digital Content 1, Supplemental Digital Content, https://links.lww.com/MD/R188; review of results, which demonstrates detailed results and interpretations). Unexpectedly, biallelic mutations in arylsulfatase A (c.1107 + 1G>C [splicing] and c1114C>T [p.Arg372Trp]) were found in case 1 which was sufficient for the diagnosis of metachromatic leukodystrophy and thereby excluded MSA. Consequently, this pair of cousins could not be classified as familial MSA.

## 5. Discussion

Neuropathologically-proven cases of familial MSA, including families with both MSA-P and multiple system atrophy-cerebellar type presentations, have been reported.^[2,3]^ This precedent made the initial consideration of familial MSA in our phenotypically divergent cousins clinically reasonable. However, the diagnosis of MSA relies predominantly on clinical criteria and documentation of an alternate condition is not easy to fully evaluate in an average clinical setting. There is a myriad of genetic, nongenetic, and immunologic conditions that can mimic MSA,^[5]^ and genetic disorders such as Perry syndrome (dynactin-1 mutation), X-linked adrenoleukodystrophy (ATP-binding cassette subfamily D, number 1 mutation), cerebrotendinous xanthomatosis (cytochrome P450 27A1 mutation) can present MSA-like clinical features, especially in early stages.

Without the genetic analysis, the diagnosis of MSA-P in case 1 would likely have been maintained, and the pair might have been misclassified as familial MSA. Various conditions, including late-onset metachromatic leukodystrophy, can present very much like MSA and therefore more caution is called for in making the diagnosis of MSA. Especially when a familial case of MSA is suspected, extensive work-ups, including a comprehensive genetic evaluation, can help to exclude alternative conditions.

## Acknowledgments

Whole genome sequencing was supported by Core G (PI: Matthew Farrer) and was funded as part of the MSA Coalition (PI: Vikram Khurana). De-identified data is made available through the MSA Genome Browser (PI: Peter Park).

## Author contributions

**Conceptualization:** Chanhee Jeong, Matthew Farrer, Vikram Khurana, Han-Joon Kim.

**Data curation:** Vikram Khurana.

**Formal analysis:** Matthew Farrer, Vikram Khurana.

**Investigation:** Chanhee Jeong, Matthew Farrer.

**Methodology:** Chanhee Jeong.

**Project administration:** Han-Joon Kim.

**Supervision:** Han-Joon Kim.

**Writing – original draft:** Chanhee Jeong.

**Writing – review & editing:** Matthew Farrer, Vikram Khurana, Han-Joon Kim.

## Supplementary Material


