# Supplementary material for: A case report of multiple system atrophy-mimics: Importance of comprehensive evaluation in suspected familial cases
Source: Medicine (Baltimore). 2026 Jan 23;105(4):e47266. doi: 10.1097/MD.0000000000047266 (PMC12851762; doi:10.1097/MD.0000000000047266)
Supplement: Supplementary file 1 [file medi-105-e47266-s001.docx]

**Supplemental Digital Content 1**

**Supplemental Digital Content to A Case Report of Multiple System Atrophy-Mimics: Importance of Comprehensive Evaluation in Suspected Familial Cases, by Chanhee Jeong, MD**

**(A) Test methods**

Whole genome sequencing was performed by the UF Clinical Genomics Program. Samples and data are processed and stored according to HIPAA compliance requirements following CAP guidelines^1^ and CLIA standards for quality and competence^2^ at UF Health Medical Laboratories. This test has been benchmarked using the National Institute of Standards and Technology ‘Genome in a Bottle’ Consortium standards (HG002 son, HG003 & HG004 parental genomes^3^). WGS precision metrics have been explored from 5-30× depth for the entire genome and results are concordant with the NIST/PrecisionFDA data “truth sets” ^4–7^ with > 95.2% analytical sensitivity and > 97.3% precision.

DNA is extracted from whole blood using the Qiagen QIAmp DNA Mini Kit. DNA is quantified by fluorescence on an Invitrogen Qubit Fluorometer. Individually-indexed genomic libraries are prepared using dual unique indexes from ~200ng DNA/individual (New England Biolabs NEBNext^®^ Ultra^TM^ II DNA Library Prep Kit for Illumina^®^). Genome library quality and quantity were confirmed by automated electrophoresis on an Agilent 2100 bioanalyzer and by qPCR. Individual libraries were normalized and pooled in equimolar ratios for 2× 150 bp paired-end sequencing at 35× depth on an Illumina NovaSeq 6000. To expedite and enable innovation, speed, data and code sharing, and to maintain security and PHI/HIPAA compliance, versioned bioinformatic pipelines for clinical genome variant calling have been developed in AWS^8^ in a containerized compute environment. Bioinformatic analyses include index deconvolution of pooled samples, read trimming, alignment, QC analyses, variant calling and annotation, with gene set panel filtering followed by variant prioritization by expert review.^9–11^ In brief, .fastq reads are aligned to the human genome reference (GRCh37/hg19) and variants are called and annotated using ‘versioned’ open source softwares including: TrimmomaticPE 0.39^12^, FastQC 0.11.9, MultiQC 1.9^13^, BWA MEM 0.7.17-r1188^14^, samtools 1.10^15^, Picard 2.23.8^16^, Strelka2 2.9.10^17^, bcftools 1.10.2^18^, snpEff 5.0c^19^, ExpansionHunter 4.0.2^20^ and cn.mops 1.8.0^21^. Computation is optimized by Nextflow.^22^

Quality control reports are generated and examined for all individual samples and batched runs, including general statistics on WGS coverage per sample, on mapping quality and on the proportion of reads surviving that process. These data quantify sequence read counts (unique, duplicate and overrepresented %), quality (Phred scores across reads, per sequence quality scores, length distributions, GC content and ‘N’ scores). Aligned files (.bam, .bai) and annotated variant files include quality scores, read orientations and depths.

Per sample variability is documented as a composite variant call file (VCF) that includes all intergenic regions, intronic variants, downstream and upstream gene variants, non-coding, missense, nonsense and silent/synonymous variants, frameshift, stop gain, splice, disruptive inframe deletions and duplications, start loss, stop loss and gene fusions. Our annotation approach is exact, comprehensive^23–25^ and includes CADD^26^ and Revel scores^27^, gnomAD frequencies^28^, ClinVar^29^ and OMIM entries.^30^

All ~22,000 genes that make up the human genome are sequenced. Reporting is restricted to exonic nonsynonymous and splicing (± 20 bp) substitutions. Only variants with > 10× coverage are reported. The clinical significance of the filtered variants is assessed according to the ACMG recommendations.^9^ Variants unrelated to the primary indication for referral were NOT intentionally searched. However, it is possible that such variants are incidentally identified during the analysis process. Incidental findings are only reported if variants are interpreted as pathogenic or likely pathogenic and if the patient consented to the return of incidental findings. Confirmation of any pathogenic mutation is achieved by orthogonal CAP-accredited testing (e.g. Sanger sequencing).

**(B) Review of results**

The mean depth of coverage for whole genome sequencing in Case 1 was 43× (bases ≥ 30× 89%) and for Case 2 was 46x (bases ≥ 30× 94%). Human genome coordinates are given with respect to GRCh37/hg19. Biallelic mutations were identified in arylsulfatase A (*ARSA*) in Case 1. These consisted of i) heterozygous Chr22:51064363 C>G splicing variant (c.1107+1G>C, ENST00000216124) with read depth of 65 (F:R_ref, F:R_alt = 12:20, 14:19), CADD score=34, not observed in gnomAD, that is predicted to be loss-of-function, and; ii) heterozygous Chr22: 51064103 G>A missense variant (c.1114C>T, p.Arg372Trp. ENST00000216124) with read depth of 57 (F:R_ref, F:R_alt = 16:11; 17:13), CADD score=32, Revel score=0.92 and gnomAD frequency = 3.99E-06 that is predicted to be pathogenic/likely pathogenic in ClinVar. These mutations are just 260 nucleotides apart and manual inspection of read-pairs within the .bam alignment, using Integrated Genome Viewer, reveals they are biallelic ‘*in trans*’ which is consistent with autosomal recessive inheritance.

**(C) References**

1. Roy S, Coldren C, Karunamurthy A, et al. Standards and Guidelines for Validating Next-Generation Sequencing Bioinformatics Pipelines: A Joint Recommendation of the Association for Molecular Pathology and the College of American Pathologists. *J Mol Diagnostics*. 2018;20(1):4-27. doi:10.1016/j.jmoldx.2017.11.003

2. Schneider F, Maurer C, Friedberg RC. International organization for standardization (ISO) 15189. *Ann Lab Med*. 2017;37(5):365-370. doi:10.3343/alm.2017.37.5.365

3. Genome in a bottle—a human DNA standard. *Nat Biotechnol*. 2015;33(7):675-675. doi:10.1038/nbt0715-675a

4. Krusche P, Trigg L, Boutros PC, et al. Best practices for benchmarking germline small-variant calls in human genomes. *Nat Biotechnol*. 2019;37(5):555-560. doi:10.1038/s41587-019-0054-x

5. Zook JM, Hansen NF, Olson ND, et al. A robust benchmark for detection of germline large deletions and insertions. *Nat Biotechnol*. 2020;38(11):1347-1355. doi:10.1038/s41587-020-0538-8

6. Zook JM, Catoe D, McDaniel J, et al. Extensive sequencing of seven human genomes to characterize benchmark reference materials. *Sci Data 2016 31*. 2016;3(1):1-26. doi:10.1038/sdata.2016.25

7. JM Z, J M, ND O, et al. An open resource for accurately benchmarking small variant and reference calls. *Nat Biotechnol*. 2019;37(5):561-566. doi:10.1038/S41587-019-0074-6

8. Amazon Elastic Compute Cloud Documentation. https://docs.aws.amazon.com/ec2/index.html?nc2=h_ql_doc_ec2. Accessed March 18, 2021.

9. Richards S, Aziz N, Bale S, et al. Standards and guidelines for the interpretation of sequence variants: a joint consensus recommendation of the American College of Medical Genetics and Genomics and the Association for Molecular Pathology. *Genet Med*. 2015;17(5):405-423. doi:10.1038/gim.2015.30

10. Riggs ER, Andersen EF, Cherry AM, et al. Technical standards for the interpretation and reporting of constitutional copy-number variants: a joint consensus recommendation of the American College of Medical Genetics and Genomics (ACMG) and the Clinical Genome Resource (ClinGen). *Genet Med*. 2020;22(2):245-257. doi:10.1038/s41436-019-0686-8

11. Kalia SS, Adelman K, Bale SJ, et al. Recommendations for reporting of secondary findings in clinical exome and genome sequencing, 2016 update (ACMG SF v2.0): a policy statement of the American College of Medical Genetics and Genomics. *Genet Med*. 2017;19(2):249-255. doi:10.1038/gim.2016.190

12. Bolger AM, Lohse M, Usadel B. Trimmomatic: a flexible trimmer for Illumina sequence data. *Bioinformatics*. 2014;30(15):2114-2120. doi:10.1093/bioinformatics/btu170

13. Ewels P, Magnusson M, Lundin S, Käller M. MultiQC: summarize analysis results for multiple tools and samples in a single report. *Bioinformatics*. 2016;32(19):3047-3048. doi:10.1093/bioinformatics/btw354

14. Li H. *Aligning Sequence Reads, Clone Sequences and Assembly Contigs with BWA-MEM*.; 2013. http://github.com/lh3/bwa. Accessed March 18, 2021.

15. samtools(1) manual page. http://www.htslib.org/doc/samtools.html. Accessed March 18, 2021.

16. Picard Tools - By Broad Institute. https://broadinstitute.github.io/picard/. Accessed March 18, 2021.

17. Kim S, Scheffler K, Halpern AL, et al. Strelka2: fast and accurate calling of germline and somatic variants. *Nat Methods*. 2018;15(8):591-594. doi:10.1038/s41592-018-0051-x

18. bcftools. http://www.htslib.org/doc/bcftools.html. Accessed March 18, 2021.

19. Cingolani P, Platts A, Wang LL, et al. A program for annotating and predicting the effects of single nucleotide polymorphisms, SnpEff: SNPs in the genome of Drosophila melanogaster strain w1118; iso-2; iso-3. *Fly (Austin)*. 2012;6(2):80-92. doi:10.4161/fly.19695

20. Dolzhenko E, Deshpande V, Schlesinger F, et al. ExpansionHunter: a sequence-graph-based tool to analyze variation in short tandem repeat regions. Birol I, ed. *Bioinformatics*. 2019;35(22):4754-4756. doi:10.1093/bioinformatics/btz431

21. Povysil G, Tzika A, Vogt J, et al. panelcn.MOPS: Copy-number detection in targeted NGS panel data for clinical diagnostics. *Hum Mutat*. 2017;38(7):889-897. doi:10.1002/humu.23237

22. Nextflow - A DSL for parallel and scalable computational pipelines. https://www.nextflow.io/. Accessed March 18, 2021.

23. Ejigu GF, Jung J. Review on the computational genome annotation of sequences obtained by next-generation sequencing. *Biology (Basel)*. 2020;9(9):1-27. doi:10.3390/biology9090295

24. Zerbino DR, Frankish A, Flicek P. Progress, challenges, and surprises in annotating the human genome. *Annu Rev Genomics Hum Genet*. 2020;21:55-79. doi:10.1146/annurev-genom-121119-083418

25. Auton A, Abecasis GR, Altshuler DM, et al. A global reference for human genetic variation. *Nature*. 2015;526(7571):68-74. doi:10.1038/nature15393

26. Kircher M, Witten DM, Jain P, O’Roak BJ, Cooper GM, Shendure J. A general framework for estimating the relative pathogenicity of human genetic variants. *Nat Genet*. 2014;46(3):310-315. doi:10.1038/ng.2892

27. Ioannidis NM, Rothstein JH, Pejaver V, et al. REVEL: An Ensemble Method for Predicting the Pathogenicity of Rare Missense Variants. *Am J Hum Genet*. 2016;99(4):877-885. doi:10.1016/j.ajhg.2016.08.016

28. Karczewski KJ, Francioli LC, Tiao G, et al. The mutational constraint spectrum quantified from variation in 141,456 humans. *Nature*. 2020;581(7809):434-443. doi:10.1038/s41586-020-2308-7

29. Landrum MJ, Lee JM, Benson M, et al. ClinVar: Improving access to variant interpretations and supporting evidence. *Nucleic Acids Res*. 2018;46(D1):D1062-D1067. doi:10.1093/nar/gkx1153

30. Amberger J, Bocchini CA, Scott AF, Hamosh A. McKusick’s Online Mendelian Inheritance in Man (OMIM®). *Nucleic Acids Res*. 2009;37(SUPPL. 1):D793. doi:10.1093/nar/gkn665
